# EBV‐Driven ODC1 Upregulation Enhances Polyamine Anabolism to Promote Viral Replication and Cisplatin Resistance in Nasopharyngeal Carcinoma

**DOI:** 10.1002/advs.77204

**Published:** 2026-08-15

**Authors:** Yueshuo Li, Xudong Hu, Chenxing Yang, Na Liu, Lin Wang, Xi Cai, Min Tang, Ya Cao, Li Shang, Feng Shi

**Affiliations:** ^1^ Department of Pathology Xiangya Hospital Central South University Changsha China; ^2^ National Clinical Research Center for Geriatric Diseases Xiangya Hospital Central South University Changsha China; ^3^ Key Laboratory of Carcinogenesis and Cancer Invasion of Chinese Ministry of Education Cancer Research Institute Xiangya School of Basic Medical Sciences Central South University Changsha China; ^4^ Molecular Imaging Research Center of Central South University Changsha Hunan China

**Keywords:** cisplatin resistance, eIF5A hypusination, Epstein‐Barr virus, innate immune responses, nasopharyngeal carcinoma, ornithine decarboxylase 1, polyamine metabolism

## Abstract

Epstein‐Barr virus (EBV) is a key oncogenic driver of nasopharyngeal carcinoma (NPC) and is closely associated with cisplatin resistance, but its roles in metabolic reprogramming and chemoresistance remain unclear. This study aimed to investigate the role of EBV in polyamine metabolic reprogramming and its underlying mechanism in mediating cisplatin resistance in NPC. Metabolomic and Metabolic Flux Analysis confirmed that EBV significantly enhances polyamine anabolism in NPC cells, with ODC1, the rate‐limiting enzyme of polyamine biosynthesis, transcriptionally upregulated by EBV‐BZLF1 via direct binding to its promoter. Functional experiments revealed that the ODC1‐spermidine axis promotes EBV replication and cell proliferation via eIF5A hypusination by upregulating EBV‐EAD and host TRAF1, respectively, and induces B‐to‐Z DNA transition to attenuate cGAS‐STING‐mediated innate immune responses. Clinically, high ODC1 expression was an independent prognostic marker for poor survival in NPC patients. In vitro and in vivo, ODC1 knockdown or pharmacological inhibition with DFMO effectively restored cisplatin sensitivity in EBV‐positive NPC cells, likely by reducing Z‐DNA formation and potentiating cisplatin‐induced innate immune responses. Collectively, our findings identify a novel EBV‐ODC1‐polyamine regulatory axis that promotes viral replication and confers a survival advantage to cancer cells, highlighting ODC1 as a promising therapeutic target to improve cisplatin efficacy in EBV‐positive NPC.

## Graphical abstract

EBV‑BZLF1 initiates ODC1‑driven polyamine anabolism that correlates with poor clinical prognosis in nasopharyngeal carcinoma. The ODC1–spermidine axis promotes viral replication, cell proliferation and innate immune evasion. Pharmacological inhibition of ODC1 rescues cisplatin sensitivity, establishing ODC1 as a viable therapeutic target for EBV‑positive malignancies.

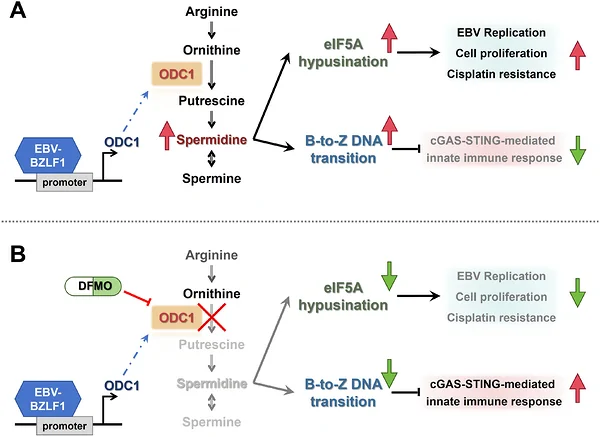

## 1 Introduction

Epstein‐Barr virus (EBV) is the first human oncogenic virus identified as capable of establishing lifelong persistence. EBV is associated with over 350 000 cases annually and accounts for 1.9% of the global cancer burden, including Burkitt, Hodgkin, post‐transplant, and T/NK cell lymphomas, as well as gastric and nasopharyngeal carcinomas (NPC) [1]. EBV has two distinct phases in its life cycle: latency and lysis. In NPC, EBV is known to remain in a type II latent infection state, which expresses key oncoproteins EBNA1, LMP1, LMP2A. Decades of extensive research have elucidated how EBV latent genes drive NPC carcinogenesis by conferring critical growth and survival advantages. EBV lytic reactivation has also been linked to the development of EBV‐associated cancers. Spontaneous lytic reactivation of EBV can be observed in NPC tumor tissues and cell lines. More than 40% of NPC samples express the immediate‐early gene BZLF1, which transcriptionally activates the lytic cycle [2]. Epidemiological evidence has confirmed that high antibody titers against EBV lytic proteins in the peripheral blood are powerful predictive markers for the development of NPC. The tumor‐associated microenvironment induced by EBV lytic reactivation promotes NPC angiogenesis, invasion, and migration [3]. The specific mechanisms by which EBV's latent and lytic cycles contribute to the tumorigenesis of epithelial malignancies remain to be fully elucidated.

Metabolic reprogramming is one of the core features of tumor cells [4]. To meet the aberrant demands for energy and biosynthetic building blocks that sustain rapid proliferation, tumor cells efficiently acquire key metabolites (ATP, nucleic acids, lipids, and amino acids) by reprogramming metabolic pathways, including glycolysis and glutaminolysis [5]. Metabolic reprogramming caused by viral infection represents a key mechanism underlying viral carcinogenesis, as viruses lack a complete enzymatic set and thus rely on the host cellular metabolic system. EBV induces metabolic remodeling in host cells to furnish the energy and biomolecules requisite for its infection and replication. Specifically, EBV‐LMP1 triggers metabolic reprogramming by targeting crucial cellular metabolic enzymes, including Hexokinase 2 (HK2), lactate dehydrogenase A (LDHA), fumarate hydratase (FH), and isocitrate dehydrogenase 2 (IDH2) [6, 7, 8, 9, 10]. Additionally, EBV can modify mitochondrial function by regulating the conformation of the ADP/ATP transporter, adenine nucleotide translocase (ANT), to maintain the balance between cellular oxidative phosphorylation and glycolysis [11]. Despite these findings, the precise mechanisms by which EBV drives metabolic reprogramming in host cells remain incompletely understood.

Polyamines, including putrescine, spermidine, and spermine, are widely involved in various cellular physiological processes [12]. In the polyamine synthesis pathway, arginine is first catalyzed by arginase 1 (ARG1) to generate ornithine, a key precursor of polyamine. Ornithine is then decarboxylated by the rate‐limiting enzyme ornithine decarboxylase 1 (ODC1) to produce putrescine, which is further converted to spermidine by spermidine synthase (SRM), and spermine is subsequently formed from spermidine by spermine synthase (SMS) [13]. Dysregulation of polyamine metabolic pathways is closely linked to the occurrence of various diseases [14]. Notably, high levels of polyamines are associated with disease progression in neuroblastoma, liver cancer, prostate cancer, lung cancer, breast cancer, gastric cancer, and colorectal cancer [15]. Spermidine, a major polyamine, acts as the substrate for hypusination (putrescine lysine modification), a unique post‐translational modification that exclusively modifies eukaryotic translation initiation factor 5A (eIF5A) in eukaryotes. Deoxyhypusine synthase (DHPS) catalyzes the transfer of the aminobutyl group of spermidine to a specific lysine residue of eIF5A, yielding hypusinated eIF5A (eIF5A^H^) [16]. eIF5A^H^ binds to ribosomes and facilitates the elongation of nascent peptide chains when ribosomes encounter a polyproline motif (PPP)and undergo translational stalling, thus rescuing protein synthesis [12, 17]. Additionally, eIF5A^H^ is involved in a spectrum of essential cellular processes, including nuclear mRNA export, mRNA decay, cell proliferation and differentiation, autophagy, and apoptosis [16]. However, the specific role of EBV in polyamine‐mediated hypusination and its associated carcinogenic mechanisms remain unclear in NPC.

Polyamines play crucial roles in various cellular functions and are essential for viruses. During the course of evolution, viruses have evolved strategies to remodel host polyamine metabolic pathways to support their own survival and productive infection. For instance, hepatitis C virus (HCV) increases the expression of ODC1 to regulate polyamine anabolism [18]. EBV downregulates SAT1 in Burkitt lymphoma cell lines to sustain polyamine levels [19]. Kaposi's sarcoma‐associated herpesvirus (KSHV) elevates the expression of ODC1 and DHPS in host cells, dynamically regulating polyamine biosynthesis and eIF5A^H^ [20]. For host innate immunity, spermidine induces conformational changes of DNA, promoting the transition of DNA from B‐form to Z‐form, and reduces the binding affinity of foreign DNA to cGAS, which attenuates the cGAS‐STING‐mediated innate immune response and thereby enables viral immune evasion [21]. Viruses such as influenza viruses, herpes viruses, and poxviruses can induce the accumulation of cytosolic Z‐DNA. Polyamines also promote the synthesis of other metabolic molecules, such as cholesterol, which in turn affects viral primary infection [22]. Therefore, targeting polyamine biosynthesis pathways represents a promising antiviral strategy.

In this study, we identified that EBV augments polyamine anabolism via ODC1, thereby increasing intracellular spermidine to drive eIF5A hypusination and Z‐DNA formation, which ultimately enhances viral replication and oncogenesis. Notably, targeted inhibition of the ODC1‐dependent signaling axis effectively reverses cisplatin resistance in EBV‐positive NPC.

## 2 Results

### 2.1 ODC1‐Mediated Polyamine Anabolism Is Enhanced in EBV‐Positive NPC Cells

To identify metabolic changes in EBV‐infected NPC cells, we used a metabolomics approach to analyze the global metabolic alterations in EBV‐positive (HONE1‐EBV) and EBV‐negative (HONE1) NPC cells. Compared to HONE1 cells, the overall differences in HONE1‐EBV are shown in a pie chart (Figure S1A). Among these differences, 27% were attributable to organic acids. Given that amino acids are important components of organic acids, and that amino acid metabolic reprogramming is a hallmark of tumor metabolic remodeling, we performed a KEGG pathway analysis of amino acid metabolism. The results revealed high abundance scores for arginine‐related ornithine and proline metabolic pathways (Figure 1A,B). To investigate the key components of the arginine metabolic pathway, we cultured NPC cells with ^13^C‐labeled arginine and tracked the carbon flux from arginine into downstream metabolites (Figure 1C). Compared with EBV‐negative HONE1 cells, HONE1‐EBV cells incorporated significantly more ^13^C‐arginine‐derived carbon into polyamine metabolites than into the urea cycle (Figure 1C and Figure S1B). Polyamine‐targeted metabolomic profiling further confirmed that arginine, putrescine, spermidine, and spermine were significantly increased in HONE1‐EBV cells (Figure 1D). Similar metabolic alterations were observed in other EBV‐positive NPC cells (Figure S1C,D). Additionally, ARG2, ODC1, SRM, and SMOX, which are key metabolic enzymes involved in polyamine biosynthesis, were transcriptionally upregulated in EBV‐positive NPC cells (Figure 1E and Figure S1E,F). These findings suggest that the polyamine biosynthesis pathway is aberrantly activated in EBV‐positive NPC cells.

**FIGURE 1:**
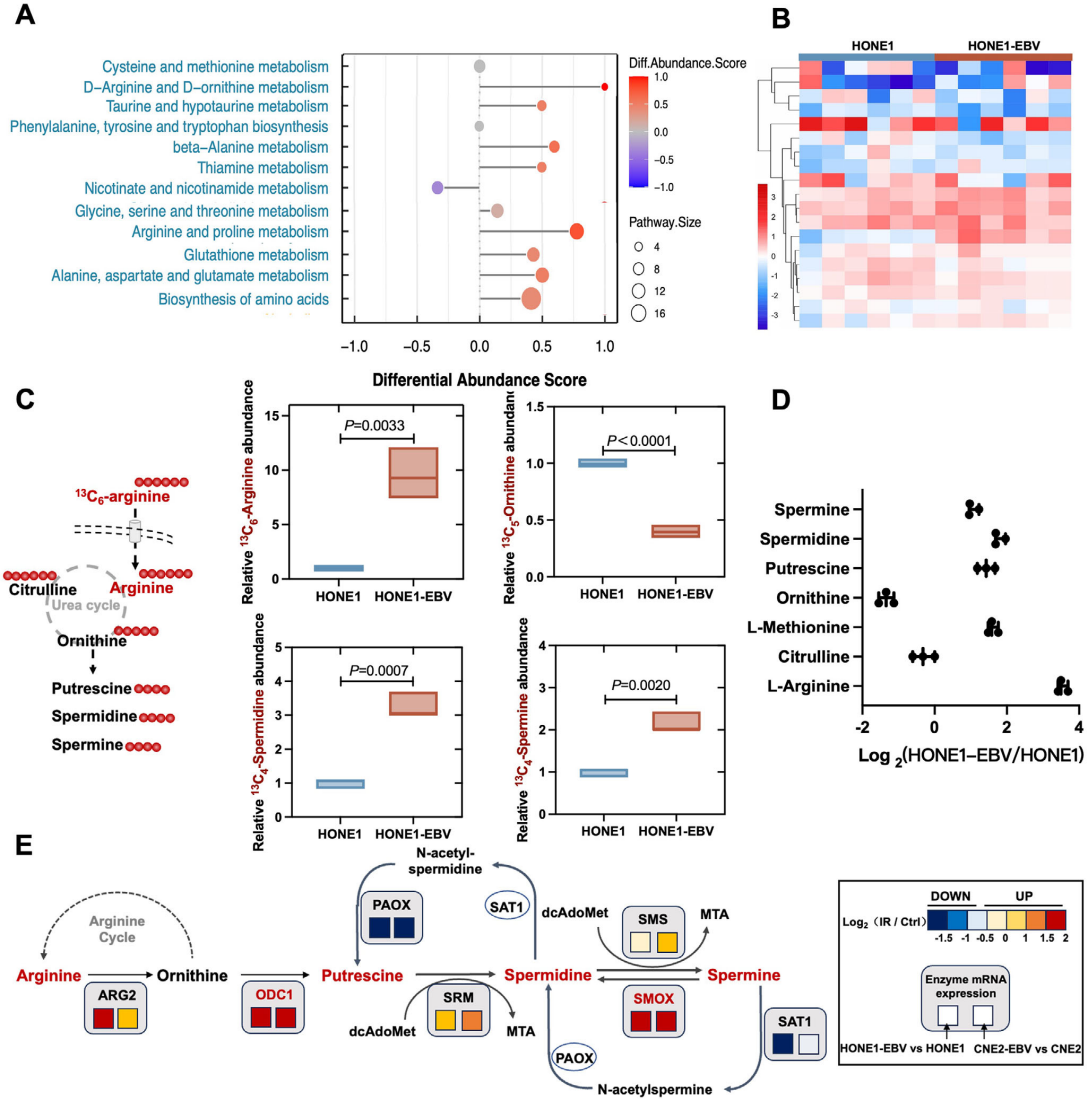
Polyamine metabolism is enhanced in EBV‐positive NPC cells. (A) Metabolic pathway KEGG enrichment analysis of differential metabolites in HONE1 and HONE1‐EBV cells. (B) Heat map of arginine metabolism‐related metabolites in HONE1 and HONE1‐EBV cells. Six biological replicates were tested for each cell line. Relative fold changes of each metabolite in individual samples are represented as the relative average increase (red) and decrease (blue). (C) NPC cells were incubated in ^13^C_6_‐arginine (20 µm) for 24 h. Metabolites were extracted and analyzed as described in Materials and Methods. Data represent means ± SD of *n* = 3 for each group. (D) Relative abundance of metabolites from polyamine‐targeted metabolomics analysis. (E) Schematic representation of polyamine metabolism. Boxes below the enzymes indicate changes in mRNA levels in EBV‐positive NPC cells compared with EBV‐negative NPC cells, respectively. mRNA expression of the main polyamine metabolic enzymes in HONE1/ HONE1‐EBV (left) and CNE2/ CNE2‐EBV cells (right) was detected by qRT‐PCR (*n* = 3). Color coding is according to log_2_‐fold change levels, as indicated.

ODC1 serves as the key rate‐limiting enzyme in polyamine biosynthesis, as it catalyzes the production of putrescine, an essential precursor for spermidine and spermine (Figure 2A). Given that ODC1 was significantly upregulated in EBV‐positive NPC cells, we constructed ODC1‐knockdown cell lines (Figure 2B–D). Cellular spermidine levels were decreased following ODC1 knockdown, and treatment with the ODC1 enzyme inhibitor DFMO (2‐difluoromethylornithine) elicited the same effect (Figure 2E,F). To further explore the role of ODC1 in arginine‐polyamine metabolic flux, a ^13^C‐arginine tracing experiment was performed. Consistent with our previous findings, we observed that spermidine and spermine levels were significantly decreased after DFMO treatment, whereas citrulline levels were increased (Figure 2G). These results indicate that ODC1 enhances polyamine biosynthesis in EBV‐positive NPC cells, and ODC1 inhibition shunts ornithine toward citrulline production and subsequent flux through the urea cycle.

**FIGURE 2:**
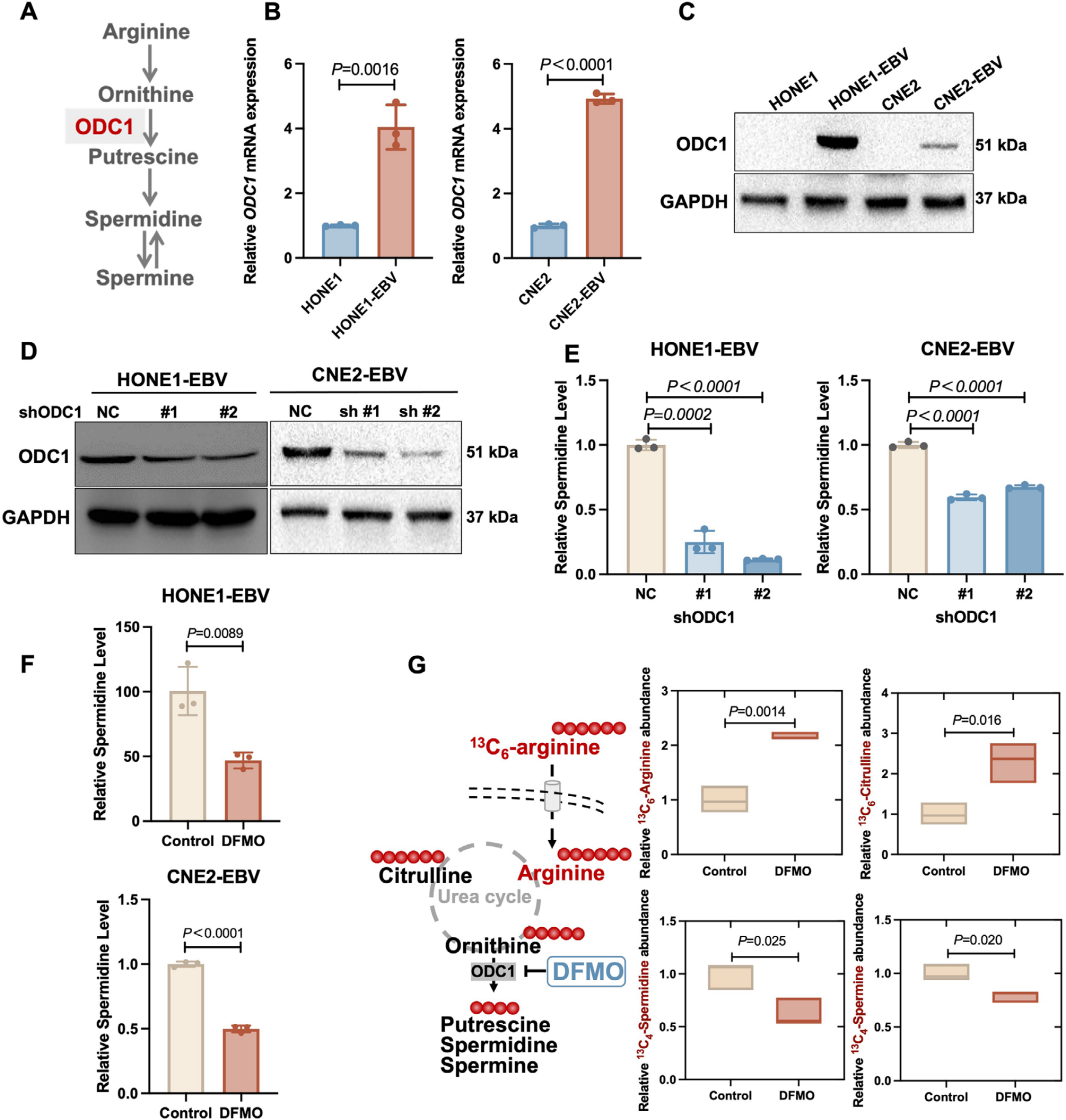
High ODC1 expression enhances polyamine biosynthesis in EBV‐positive NPC cells. (A) Schematic diagram of polyamine biosynthesis metabolism. (B) mRNA expression of ODC1 in NPC cells was detected by qRT‐PCR. (C) Immunoblot analysis of ODC1 expression in NPC cells. GAPDH served as the loading control. (D) NPC cells were transfected with ODC1 shRNAs, followed by cell lysate extraction and Western blot analysis. GAPDH served as the loading control. (E) Spermidine levels in NPC cells were measured by ELISA upon ODC1 knockdown. (F) Spermidine levels in NPC cells were measured by ELISA with or without DFMO (10 µm) treatment for 48 h. (G) HONE1‐EBV cells were treated with or without DFMO (10 µm) for 24 h, then incubated in ^13^C_6_‐arginine (20 µM) for another 24 h. Metabolites were extracted and analyzed as described in Materials and Methods. Data represent means ± SD of *n* = 3 for each group.

### 2.2 EBV‐encoded BZLF1 Facilitates the Transcriptional Activation of ODC1

To further investigate the regulatory mechanism of ODC1 in the context of EBV infection, GSEA analysis was performed using the head and neck squamous cell carcinoma (HNSCC) dataset from the LinkedOmics database. These results revealed a strong positive correlation between EBV infection and ODC1 expression (Figure 3A). Next, we analyzed public RNA sequencing data of 113 NPC patients (GSE102349). ODC1 mRNA levels were significantly positively associated with a high EBV activity score (Figure 3B). We further assessed the association between ODC1 and a panel of EBV genes. Among all detected EBV transcripts, only BZLF1, an EBV‐encoded transcriptional transactivator, showed a significant positive correlation with ODC1, whereas no such correlation was observed for other EBV‐encoded genes (Figure 3C and Figure S2). Ectopic overexpression of BZLF1 in EBV‐negative NPC cells markedly elevated ODC1 at mRNA and protein levels (Figure 3D,E). In a time‐course experiment with TPA treatment, gradual BZLF1 induction coincided with continuous increases in ODC1 expression and cellular spermidine levels (Figure 3F and Figure S3A). Further dose‐gradient transfection validated positive dose‐dependent regulation of ODC1 expression by BZLF1 (Figure S3B).

**FIGURE 3:**
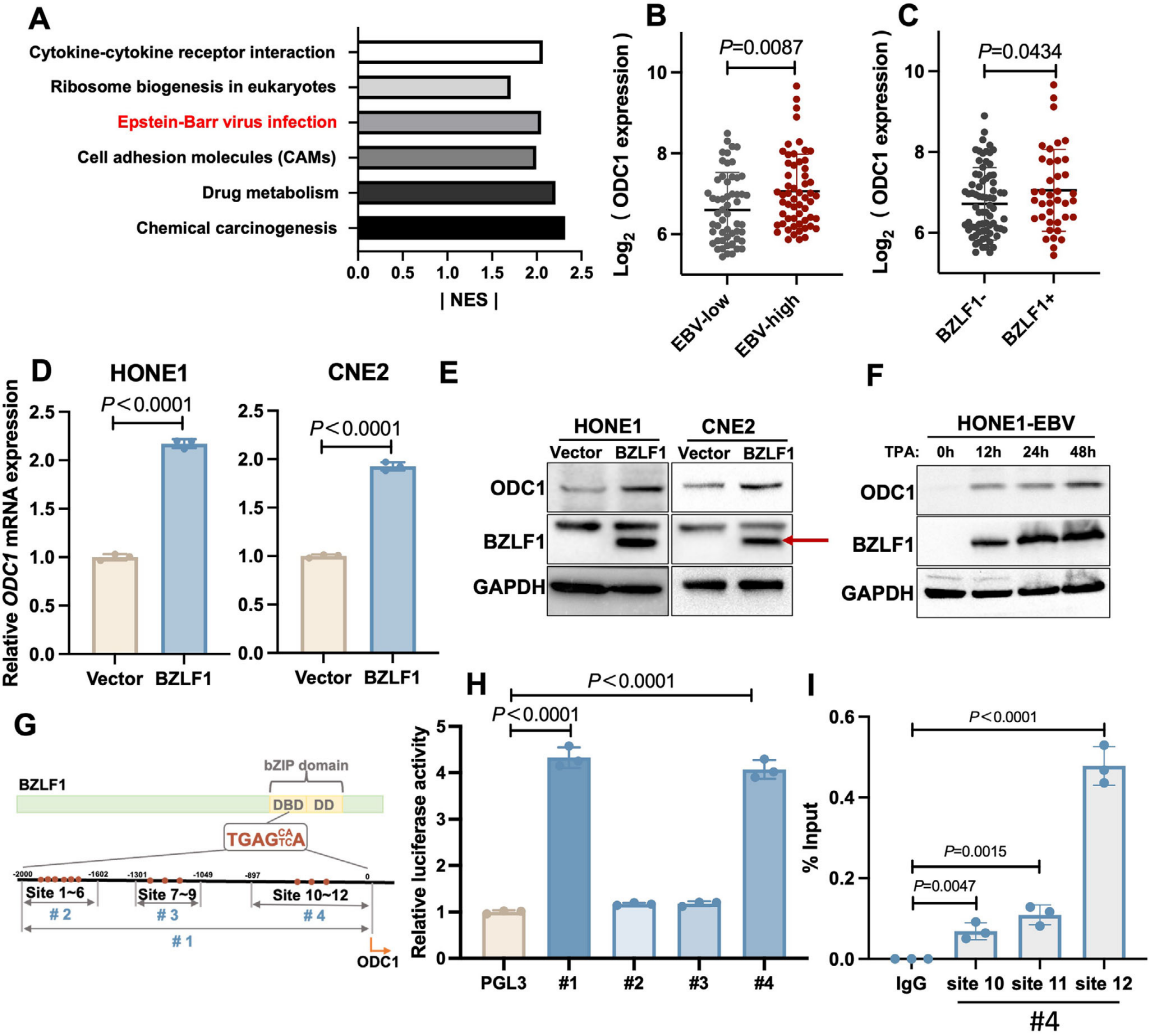
EBV‐BZLF1 promotes ODC1 transcriptional activation. (A) GSEA analysis of ODC1‐related pathways in the HNSCC dataset from the LinkedOmics database. (B) ODC1 expression levels were calculated relative to EBV activity score. Samples from the GSE102349 dataset were stratified into high and low EBV activity groups based on the median value of the EBV activity score. (C) ODC1 expression levels were calculated relative to BZLF1 gene expression. Groups with high and low BZLF1 levels were defined according to the median expression from GSE102349. (D,E) HONE1 and CNE2 cells were transfected with empty vector (Vector) or BZLF1‐expressing plasmids; ODC1 mRNA expression levels were analyzed by quantitative PCR (D), and cell lysates were subjected to Western blot analysis (E). GAPDH served as the loading control. (F) HONE1‐EBV cells were treated with TPA (10 µm), and cell lysates were collected at the indicated time points for Western blot analysis. GAPDH served as the loading control. (G) Schematic illustration of the *ODC1* promoter depicting potential BZLF1 binding sites (Orange dots). (H) 293T cells were co‐transfected with BZLF1 and luciferase reporter plasmids driven by the wild‐type or mutant ODC1 promoter (#1‐#4 domain as shown in E), along with the PLR‐TK construct. Error bars, SD. (I) HONE1‐EBV cells were treated with TPA (10 µm) for 24 h, and then level of BZLF1 binding to the ODC1 promoter was analyzed using ChIP followed by RT‐PCR of three specific regions (*n* = 3).

To verify whether BZLF1 directly transcriptionally regulates ODC1 expression, promoter analysis identified twelve putative BZLF1‐binding sites (TGAGTCA or TGAGCAA) within the ODC1 promoter region (Figure 3G). The promoter region was divided into four reporter plasmids (−2000–0 bp, −2000–1602 bp, −1301–1049 bp, and −897–0 bp) according to these predicted binding sites. Dual‐luciferase assays revealed that the reporter construct containing the 10–12 binding site region alone was sufficient to drive luciferase activity (Figure 3H). ChIP‐qPCR results revealed obvious BZLF1 enrichment at sites 10, 11, and 12 after TPA treatment, with site 12 showing the strongest binding (Figure 3I), suggesting that site 12 serves as the predominant functional binding motif responsible for BZLF1‐driven ODC1 transcription.

### 2.3 The ODC1‐Spermidine Pathway Contributes to EBV DNA Replication

To further investigate the advantages that EBV gains from reprogramming polyamine metabolism, we first tested EBV replication, using EBV DNA copies as a key marker of EBV‐related tumors. After ODC1 knockdown, EBV DNA copies were significantly decreased (Figure S4A,B). Furthermore, we used TPA, a classic inducer of EBV replication, and found that ODC1 knockdown or enzymatic activity inhibition abrogated TPA‐induced EBV DNA replication (Figure 4A,B). Similarly, EBV‐BZLF1 expression‐triggered EBV replication was also abrogated by ODC1 knockdown or enzymatic activity inhibition (Figure S4C,D). Spermidine supplementation effectively rescued the impairment of EBV replication caused by ODC1 knockdown (Figure 4C). These results confirm that EBV reprograms host polyamine metabolism to support its replication.

**FIGURE 4:**
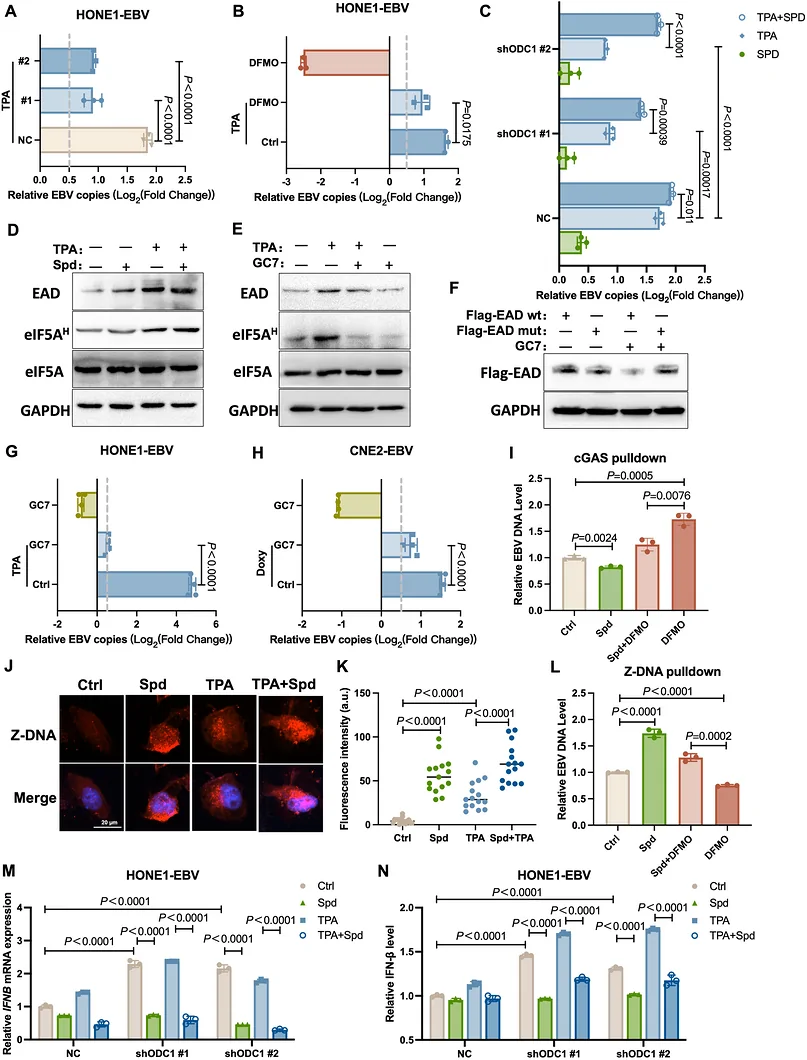
The ODC1‐spermidine pathway contributes to EBV DNA replication and immune evasion. (A) Real‐time PCR analysis of EBV DNA copy numbers after TPA treatment (10 µm, 24 h) in ODC1‐knockdown HONE1‐EBV cells. (B) HONE1‐EBV cells were pretreated with DFMO for 24 h, followed by TPA treatment (10 µm, 24 h). EBV DNA copy numbers were calculated by real‐time PCR. (C) HONE1‐EBV cells were infected with ODC1 lentiviral shRNAs and subsequently treated with the indicated agents (TPA: 10 µM; Spermidine (Spd): 10 µm) for 24 h. EBV DNA copy numbers were quantified by real‐time PCR after the indicated treatments. (D) HONE1‐EBV cells were treated with TPA (10 µm) or spermidine (10 µm) for 24 h, and cell lysates were subjected to Western blot analysis. (E) HONE1‐EBV cells were treated with TPA (10 µm) or GC7 (10 µm) for 24 h, and cell lysates were subjected to Western blot analysis. (F) HONE1 cells were transfected with wild‐type EAD (Flag‐EAD wt) or poly‐proline mutant‐EAD (Flag‐EAD mut) plasmids, and then treated with GC7 (10 µm) for 24 h. Cell lysates were subjected to Western blot analysis. (G) HONE1‐EBV cells were pretreated with GC7 (5 µm) for 24 h, followed by TPA treatment (10 µm, 24 h). EBV DNA copy numbers were calculated by real‐time PCR. (H) CNE2‐EBV cells were pretreated with GC7 (10 µm) for 24 h, followed by Doxy treatment (10 µm, 24 h). EBV DNA copy numbers were calculated by real‐time PCR. (I) ChIP analysis of EBV genomic DNA binding to cGAS in HONE1‐EBV cells with indicated treatments. Anti‐cGAS antibody was used, and quantitative real‐time PCR was performed to evaluate the binding level of EBV genomic to cGAS. (J) NPC cells were stained for Z‐DNA after the indicated treatments. Scale bar: 20 µm. (K) Quantification of Z‐DNA fluorescence signal intensity. (L) ChIP analysis was performed in NPC cells following the indicated treatments. Anti‐Z‐DNA antibody was used, and quantitative real‐time PCR was performed to evaluate EBV genomic in Z‐DNA. (M) The expression of *IFNB* mRNA in cells with the indicated treatments was measured by quantitative PCR, NC: negative control. N: The production of IFN‐β protein was assayed by ELISA. The relative production of IFN‐β was calculated. NC: negative control.

A key function of spermidine is to act as the substrate for hypusine post‐translational modification. Cumulative evidence has demonstrated that spermidine‐mediated eIF5A hypusination plays an important role in regulating viral protein expression and DNA replication [13]. eIF5A^H^ can restore translation when ribosomes encounter continuous polyproline motifs, thereby enhancing protein synthesis. Therefore, we analyzed the amino acid sequences of EBV‐encoded proteins and found that EBV DNA polymerase synthesis factor EAD (Early Antigen Diffuse) contains typical polyproline motifs. As a core multifunctional protein in the EBV replication process, EAD acts as an accessory protein for viral DNA polymerase and significantly enhances viral DNA synthesis. In NPC tissue microarrays, EAD protein levels were positively correlated with ODC1 expression (Figure S4E,F). Spermidine supplementation increased EAD protein expression, while treatment with the hypusination inhibitor GC7 abrogated TPA‐induced EAD expression (Figure 4D,E). We further constructed an EAD mutant with polyproline residues mutated (Table S2). The mutant was resistant to GC7 inhibition (Figure 4F), confirming that polyproline motifs are essential for eIF5A^H^‐dependent EAD translation. EBV DNA copy number analysis also showed that GC7 significantly inhibited EBV DNA replication (Figure 4G,H). These results indicate that spermidine‐mediated hypusination contributes to the expression of EBV replication‐associated viral proteins and subsequent viral DNA replication.

### 2.4 Elevated Spermidine Levels Enable EBV Immune Evasion

High levels of viral DNA in host cells greatly increase the risk of recognition by the host immune system. Notably, another important function of polyamines is that spermidine promotes the conformational conversion of DNA from B‐form to Z‐form, thereby reducing the binding affinity of foreign DNA to cGAS and blunting the cGAS‐STING‐mediated innate immune response, which facilitates viral immune evasion [21]. cGAS ChIP‐qPCR revealed that spermidine significantly suppressed the binding of EBV genomic DNA to cGAS, and this inhibitory effect was fully reversed by DFMO treatment (Figure 4I). Consistent with this finding, spermidine robustly induced Z‐DNA conformational transition in EBV‐positive NPC cells, even under replication‐inducing conditions (Figure 4J,K). Moreover, spermidine facilitated the conversion of EBV DNA to the Z‐form, which was abolished by DFMO (Figure 4L). We next measured the production of IFN‐β, a canonical activation marker of the STING pathway. ODC1 knockdown significantly enhanced IFN‐β production, and spermidine supplementation effectively decreased IFN‐β levels. Besides, activation of the EBV replication cycle markedly increased IFN‐β production in ODC1‐knockdown cells, while spermidine supplementation effectively reversed this effect (Figure 4M,N and Figure S4G). These results confirm that enhanced polyamine anabolism facilitates EBV replication and enables evasion of host innate immune surveillance.

### 2.5 The ODC1‐Spermidine‐eIF5A^H^ Axis Promotes Cancer Cell Proliferation

Polyamines are critical for cell growth and are highly abundant in various cancers. Since EBV is a key driver of NPC, this highlights the potential functional importance of the EBV‐driven polyamine biosynthesis in tumorigenesis. To validate this hypothesis, we assessed cell proliferation by EdU and CCK‐8 assays. As expected, both knockdown and pharmacological inhibition of ODC1 effectively decreased the viability of EBV‐positive NPC cells (Figure 5A–E and Figure S5A–D). Furthermore, supplementation with spermidine, the most abundant polyamine in EBV‐positive NPC cells, effectively reversed the effects of ODC1 knockdown or pharmacological inhibition. These findings suggest that the EBV‐driven polyamine biosynthesis is critical for cell proliferation (Figure 5F,G and Figure S5E,F). To examine whether eIF5A^H^ contributes to polyamine‐mediated cell proliferation, we treated cells with the hypusination inhibitor GC7 (Figure 5C). GC7 treatment significantly inhibited cell viability, which could be reversed by spermidine (Figure 5H and Figure S5G). Similarly, GC7 abrogated cell proliferation driven by polyamine biosynthesis (Figure 5I and Figure S5H). These results indicate that polyamines promote EBV‐positive NPC cell proliferation via eIF5A hypusination. We next performed LC‐MS/MS proteomic profiling to identify differentially expressed proteins following eIF5A hypusination inhibition. Among the proteins quantified in both control and GC7‐treated cells, 90 were upregulated, and 132 were downregulated (fold change > 1.5, *p* < 0.05) (Figure 5J). KEGG pathway enrichment analysis of these differentially expressed proteins revealed significant enrichment in pathways related to viral infection and viral carcinogenesis, including the EBV infection pathway (Figure 5K). Notably, TRAF1 was a key protein significantly altered in these pathways (Figure 5L). TRAF family proteins are key adaptors bridging EBV oncoproteins to host cellular signaling pathways, particularly NF‐κB signaling. Further, we examined TRAF1 protein expression levels and found that GC7 treatment inhibited both eIF5A^H^ and TRAF1 protein expression, whereas spermidine increased eIF5A^H^ and TRAF1 protein levels (Figure 5M,N).

**FIGURE 5:**
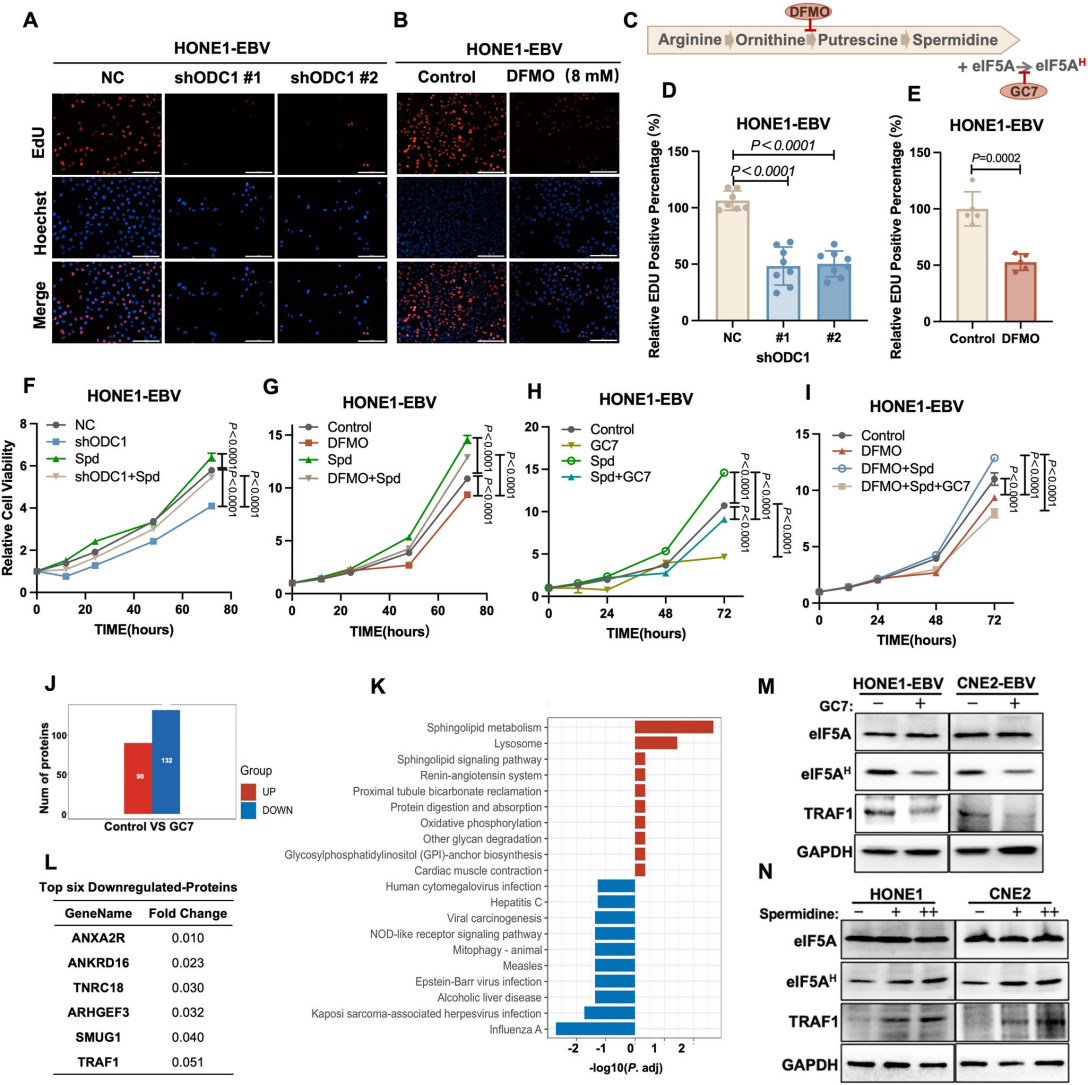
The ODC1‐spermidine‐eIF5A^H^ axis promotess cancer cell proliferation. (A,B) EdU staining analysis with shODC1 or DFMO treatment in HONE1‐EBV cells. (C) Schematic representation of polyamine metabolism. DFMO inhibits ODC1 activity, and GC7 inhibits eIF5A hypusination. (D,E) EdU fluorescence intensity with an average value above 500 was defined as positive. Nuclei were counterstained with Hoechst 33342. (Scale bar: 200 µm). (F) ODC1 knockdown HONE1‐EBV cells were treated with or without spermidine (10 µm), and cell viability was assessed by the CCK‐8 assay at the indicated time points. (G) HONE1‐EBV cells were treated with or without DFMO (10 µm) for 24 h, followed by incubation with spermidine (10 µm); cell viability was assessed by the CCK‐8 assay at the indicated time points. (H) HONE1‐EBV cells were treated with spermidine (10 µm) alone or spermidine combined with GC7 (10 µm), and cell viability was assessed by the CCK‐8 assay at the indicated time points. (I) Cell viability was assessed by the CCK‐8 assay following the indicated treatment. (J) Differential proteins in GC7 vs. control cells by proteomics analysis. (K) KEGG pathway enrichment analysis of differentially expressed proteins in GC7 vs. control cells. (L), Top six downregulated proteins in GC7 group. (M) Western blot analysis of eIF5A, eIF5A^H^, and TRAF1 protein levels in NPC cells with or without GC7 (10 µm) treatment. (N) Western blot analysis of eIF5A, eIF5A^H^, and TRAF1 protein levels in NPC cells with indicated spermidine treatment. Error bars, SD.

### 2.6 ODC1 Overexpression Positively Correlates With Poor Prognosis of NPC Patients

Having established that the EBV‐ODC1‐spermidine axis regulates NPC cell proliferation, we further examined its clinical relevance in NPC patients. Single‐cell RNA‐seq analysis identified high ODC1 expression in malignant cells (Figure 6A–C). ODC1 expression was also analyzed in pathological sections of nasopharyngitis (NP) and NPC tissues. Immunohistochemical staining showed significantly elevated ODC1 expression in NPC tissues compared with NP tissues (Figure 6D,E and Figure S6). Similarly, immunofluorescence staining revealed high ODC1 protein expression in NPC tissues, whereas adjacent noncancerous tissues exhibited low ODC1 levels (Figure 6F,G). We also conducted Kaplan‐Meier analysis using a GEO dataset of 122 NPC samples, which showed that patients with high ODC1 expression had shorter overall survival and recurrence free survival (Figure 6H). Furthermore, we divided 126 NPC samples in the tissue microarray into high and low ODC1 expression groups for Kaplan–Meier analysis, which confirmed that patients with high ODC1 expression had significantly shorter overall survival and recurrence free survival (Figure 6I). Additionally, high ODC1 expression was significantly associated with tumor recurrence and metastasis in patients (Table 1). Univariate and multivariate Cox proportional hazards regression analyses demonstrated that high ODC1 expression was an independent risk factors for poor overall survival in NPC patients (Table 2). These findings indicate that high ODC1 expression correlates with poor survival outcomes and an increased risk of recurrence and metastasis in NPC patients.

**FIGURE 6:**
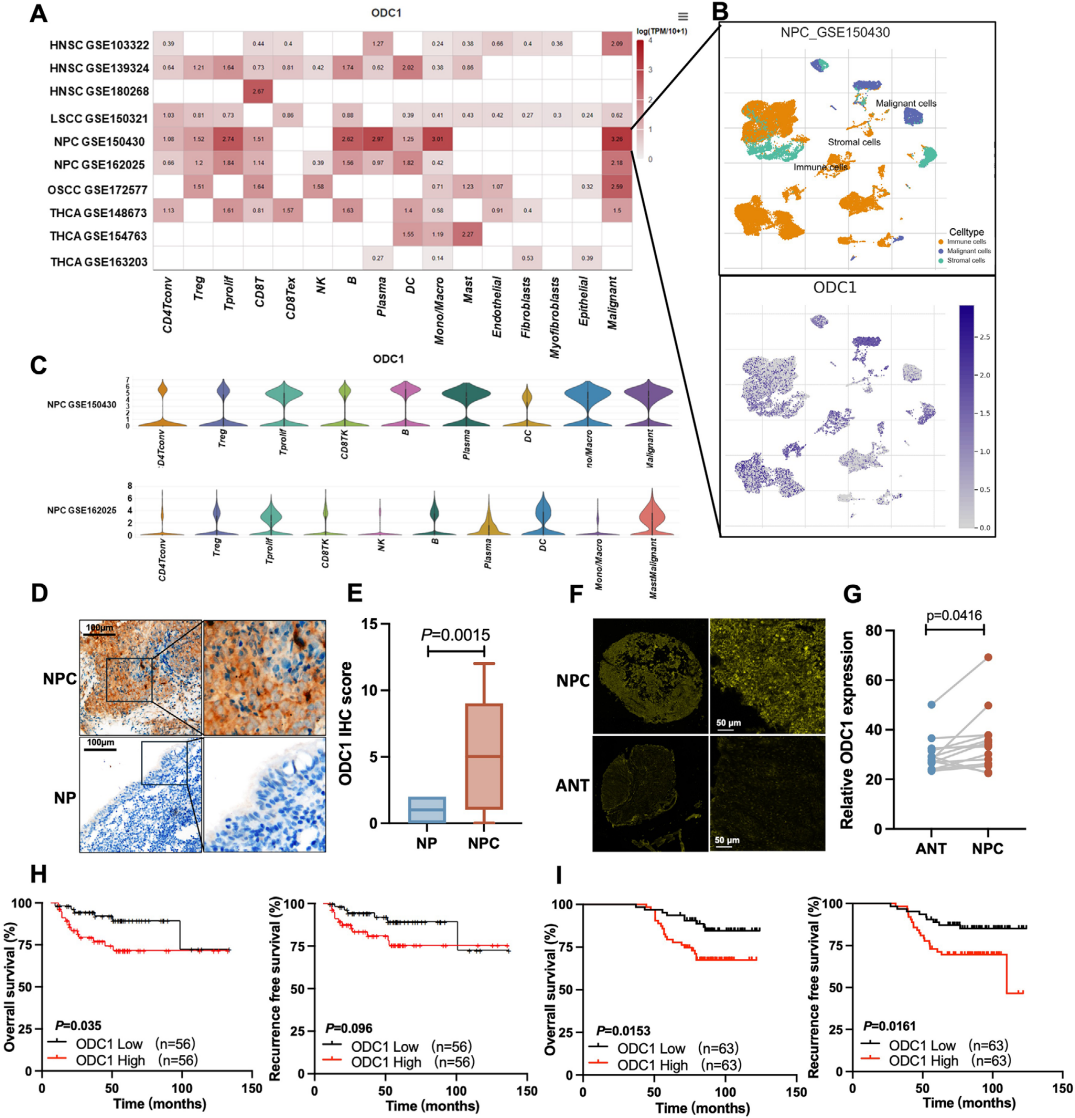
ODC1 overexpression positively correlates with poor prognosis of NPC patients. (A) ODC1 expression across different cell types was analyzed by the TISCH2 single‐cell RNA‐seq database. (B) UMAP visualization of ODC1 expression in malignant, stromal, and immune cell populations. (C) ODC1 expression in malignant, stromal, and immune cell populations. (D) Representative IHC staining of ODC1 expression in pathological sections from nasopharyngitis (NP) and NPC patients (Scale bar: 100 µm). (E) Tissue IHC scores of ODC1 expression in NPC tissues (*n* = 31) compared to NP tissue (*n* = 9). Error bars, SD. (F) Representative IHC‐F staining of ODC1 expression in a tissue microarray of NPC and adjacent noncancerous tissues (ANT) (Scale bar: 50 µm). (G) Average fluorescence intensity of ODC1 expression in ANT tissues compared with that in NPC tissues (*n* = 14). Error bars, SD. (H) Overall survival and recurrence free survival rates of NPC patients from the GSE102349 dataset with low (*n* = 56) or high (*n* = 56) ODC1 expression were estimated by the Kaplan–Meier method. Group according to ODC1 median expression. (I) Overall survival and recurrence free survival rates of NPC patients from NPC tissue microarray with low (*n* = 63) or high (*n* = 63) ODC1 expression were estimated by the Kaplan–Meier method. Group according to ODC1 median expression.

**TABLE 1:** Clinical characteristics and ODC1 expression level in 126 NPC patients.

Clinical Characteristics	ODC1 Low expression	ODC1 High expression	*p*‐value
**Age** (yr), mean ± S.D.	51.07 ± 12.04	51.04 ± 12.53	
**Gender**			0.873 ^a^
Males(n)	39	42	
Females(n)	21	24	
**Metastasis**			
Negative (n)	42	34	0.034 ^a^
Positive (n)	18	32	
**Recurrence**			0.039 ^a^
Negative	40	32	
Positive	20	34	
**Ki‐67**			
≥40%	26	48	0.001 ^a^
<40%	34	18	
**Pathologic T stage**			
T1/T2	43	34	0.020 ^a^
T3/T4	17	32	
**Lymph node metastasis**			
N0	49	41	0.015 ^a^
N1	11	25	

^a^ Pearson^,^s χ^2^ test.

**TABLE 2:** Univariate and multivariate COX regression analysis of overall survival of NPC.

Variable	Univariate analysis			Multivariate analysis		
	HR^b^	95% CI^c^	*P*	HR	95%CI	*P*
**Age** (>50 vs ≤50)	3.772	1.438–9.898	0.007^*^	2.377	0.891‐6.340	0.084
**Sex** (Female vs Male)	0.463	0.193–1.113	0.085			
**EAD** (High vs Low)	2.041	0.963–4.327	0.063			
**ODC1** (High vs Low)	2.856	1.259–6.479	0.012^*^	2.316	1.010–5.310	0.047^*^
**Stage** (III/IV vs I/II)	12.876	4.451–37.252	0.001^*^	2.983	0.478–18.636	0.242
N^a^ (N1/N2/N3 vs N0)	15.063	5.699–39.811	0.001^*^	5.060	0.939–27.272	0.059

^a^ ymph node metastases.

^b^ hazard ratio.

^c^ confidence interval.

* significant difference.

### 2.7 ODC1 Inhibition Potentiates Cisplatin Response in EBV‐Positive NPC Models

Platinum‐based agents have been reported to affect the polyamine pathway by upregulating the key catabolic gene spermine/ spermidine N1‐acetyl transferase (SSAT), and downregulating biosynthetic genes, including ODC1 and S‐adenosylmethionine decarboxylase (SAMDC), thereby leading to intracellular polyamine pool depletion [23, 24]. Notably, in cisplatin‐resistant ovarian cancer cells, ODC1 is highly expressed, and inhibition of polyamine uptake enhances cellular sensitivity to cisplatin [25, 26]. This highlights the functional importance of the polyamine pathway in chemotherapeutic responses. EBV is a crucial factor modulating chemotherapeutic efficacy, and our previous studies have demonstrated that EBV significantly increases cisplatin resistance in NPC cells by activating multiple oncogenic signaling pathways [11, 27]. Therefore, we further investigated whether targeting polyamine biosynthesis could enhance the efficacy of chemotherapy in EBV‐positive NPC cells. First, ODC1 knockdown or pharmacological inhibition with DFMO reduced the colony formation rate of EBV‐positive NPC cells and significantly potentiated the antitumor effect of cisplatin (Figure 7A,B and Figure S7A,B). In addition, combination treatment with DFMO and cisplatin significantly reduced cell viability compared with either single agent, with a combination index (CI) < 1 confirming a synergistic effect in EBV‐positive NPC cells (Figure 7C and Figure S7C–E).

**FIGURE 7:**
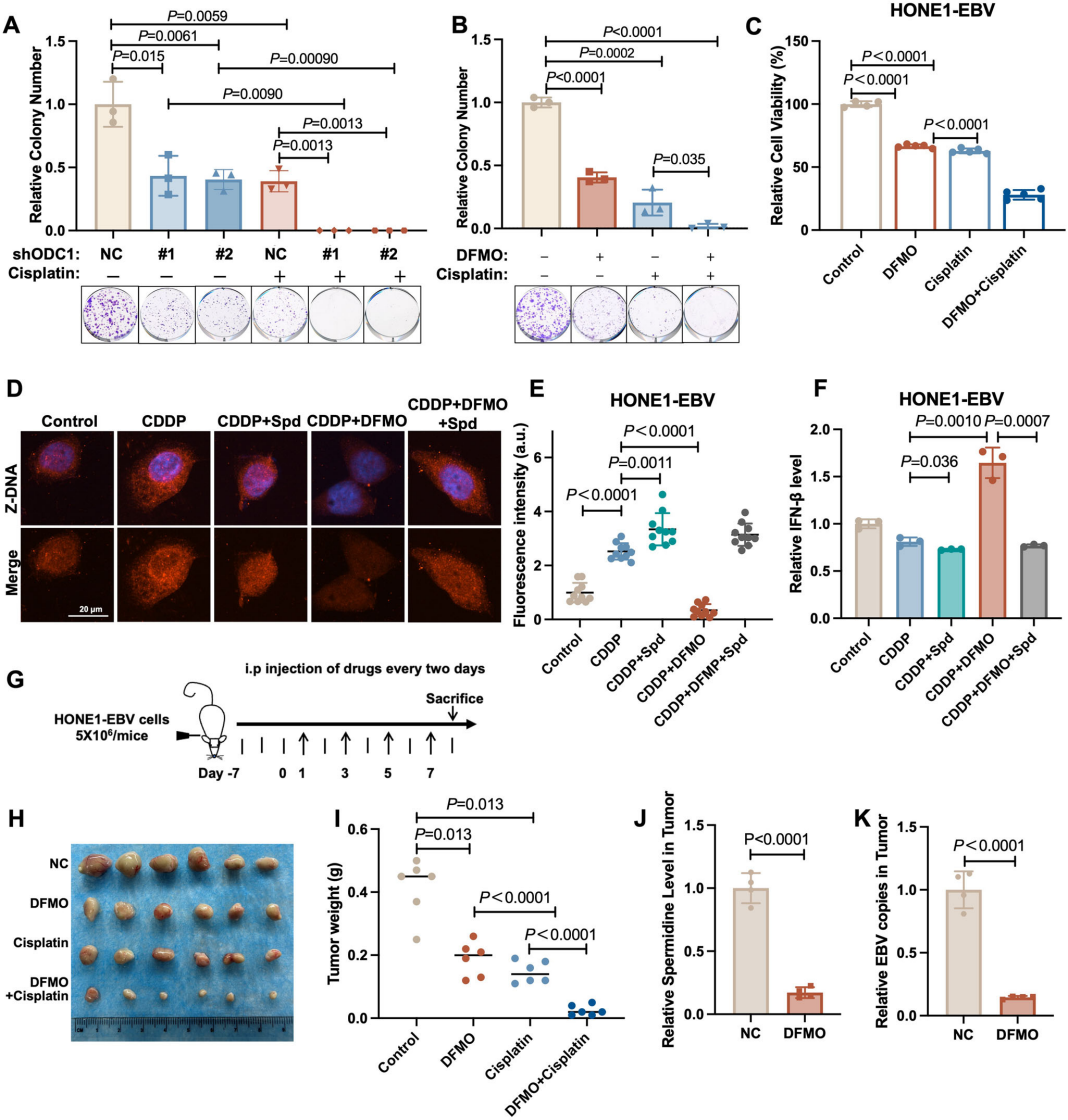
Targeting ODC1 enhances cisplatin sensitivity of EBV‐positive NPC cells in vitro and in vivo. (A) ODC1‐knockdown HONE1‐EBV cells were treated with or without cisplatin (10 µm), and cell proliferation was assessed using the colony formation assay. Relative survival fractions are shown. (B) HONE1‐EBV cells were treated with or without DFMO (10 µm) for 24 h, followed by incubation with or without cisplatin (10 µm). Cell proliferation was assessed using the colony formation assay. Relative survival fractions are shown. (C) HONE1‐EBV cells were treated with or without DFMO (10 µm) for 24 h, followed by incubation with or without cisplatin (10 µm). Cell viability was assessed using the CCK‐8 assay. (D) HONE1‐EBV cells were stained for Z‐DNA after the indicated treatments. Scale bar: 20 µm. (E) Quantification of Z‐DNA fluorescence signal intensity. (F) The production of IFN‐β protein was assayed by ELISA. The relative production of IFN‐β was calculated. (G) Schematic overview of the study design. The NPC xenograft model was established by using HONE1‐EBV cells. (H) Representative images of xenografts from different treatment groups. (I) Tumor weights of xenograft tumors with various treatments. (J) Relative IFN‐β production was quantified in xenograft tumors. (K) Relative EBV copy numbers were quantified in xenograft tumors. Error bars, SD.

The aforementioned findings confirm that EBV promotes polyamine biosynthesis to suppress cGAS‐STING‐mediated innate immune signaling in host tumor cells. Immune activation represents a critical mechanism underlying chemotherapy efficacy. For instance, cisplatin activates the STING‐IRF3‐IFN pathway in NPC cells [28, 29]. To determine whether polyamine metabolism mediates this immunosuppressive effect, we investigated its role in regulating cisplatin‐induced innate immune response. Low‐dose cisplatin treatment significantly induced Z‐DNA formation in HONE1‐EBV cells. Notably, spermidine supplementation further augmented this inductive effect. Conversely, DFMO treatment effectively reduced Z‐DNA levels, and this reduction was rescued by spermidine supplementation (Figure 7D,E). Additionally, DFMO significantly enhanced IFN‐β production upon cisplatin treatment, and this effect was reversed by spermidine (Figure 7F). To investigate whether this regulatory effect was EBV‐independent, we performed the same experiments in polyamine‐active lung adenocarcinoma and breast cancer cells, and obtained analogous results (Figure S8A–E). Thus, our results demonstrate that polyamines inhibit cisplatin‐triggered innate immune response in cancer cells. EBV hijacks this mechanism by enhancing polyamine biosynthesis, thereby driving cisplatin resistance.

Furthermore, a xenograft mouse model was used to determine whether targeting polyamine biosynthesis sensitizes EBV‐positive NPC cells to cisplatin in vivo (Figure 7G). As shown, DFMO monotherapy significantly reduced tumor volume and weight, with no significant alterations in mouse body weight (Figure 7H,I and Figure S9A,B). Histopathological analysis showed no obvious pathological damage in liver, spleen, and kidney tissues from the treatment groups (Figure S9C). Moreover, the combination of DFMO and cisplatin elicited a marked reduction in tumor volume and tumor weight compared with all other groups. DFMO treatment also significantly decreased the levels of spermidine and EBV DNA copies in the xenograft tissues (Figure 7J,K). Collectively, these results indicate that targeting polyamine biosynthesis enhances the sensitivity of EBV‐positive NPC to cisplatin.

## 3 Discussion

A large number of metabolic intermediates and products, including acetyl‐CoA, α‐ketoglutarate, and lactic acid, generated via cellular metabolic processes act not only as biosynthetic substrates but also as modification donors that participate in protein post‐translational modifications such as histone acetylation and lactylation. Catalyzed by dedicated enzymes, these metabolites form covalent linkages with specific amino acid residues of target proteins, thereby precisely regulating protein functional activity, subcellular localization, stability, and interaction networks. This process closely couples the cellular metabolic state to core biological processes including gene expression and signal transduction.

Emerging evidence suggests that even partial or abortive lytic reactivation contributes to tumor progression in EBV‐positive epithelial malignancies [3, 30]. In NPC, the viral immediate‐early protein BZLF1 plays a pivotal role: elevated BZLF1 expression correlates with poor clinical outcomes and drives aggressive, metastatic phenotypes [31]. As an immediate‐early lytic gene, BZLF1 initiates the viral lytic cascade; additionally, it can transactivate host genes to hijack cellular processes. BZLF1 directly binds to Zta‐response elements in the promoter of host genes such as *KDM5B*—an identified oncogene that promotes the progression of epithelial malignancies [32]. In the present study, we identified that BZLF1 transcriptionally upregulates ODC1 expression. Promoter analysis revealed that this regulation is mediated through twelve putative BZLF1 binding sites (consensus sequence: TGAGTCA or TGAGCAA) within the ODC1 promoter region, and the transcriptional regulatory region of ODC1 by EBV‐BZLF1 is primarily located in the −897–0 bp range. BZLF1‐mediated ODC1 upregulation enhances polyamine biosynthetic metabolism, which in turn provides the essential building blocks to facilitate viral replication. This further elucidates how EBV manipulates the host cellular and metabolic microenvironments to favor tumor survival and progression via lytic reactivation and effector proteins such as BZLF1.

eIF5A^H^ exerts its biological functions by interacting with target molecules, and emerging evidence has revealed that it also plays an important role in regulating viral protein expression and DNA replication [33]. eIF5A has been identified as a key cofactor for the Rev transport factor in human immunodeficiency virus type 1 (HIV‐1) [34]. Kaposi's sarcoma‐associated herpesvirus (KSHV) upregulates the expression of ODC1 and DHPS in host cells, and dynamically modulates polyamine biosynthesis activity and eIF5A^H^ levels. The synthesis of key latent proteins (LANA) and lytic proteins (RTA) of KSHV relies on eIF5A^H^. Targeting the polyamine biosynthetic pathway or inhibiting the formation of eIF5A^H^ can block the replication of KSHV [20, 35]. Through amino acid sequence analysis of EBV‐encoded proteins, we found that the EBV DNA polymerase synthesis factor EAD (Early Antigen Diffuse) contains canonical polyproline motifs. EAD is a core, multifunctional protein in the EBV replication process which acts as an accessory factor for viral DNA polymerase, markedly enhancing its processive DNA synthesis activity. Spermidine treatment increased EAD protein expression, whereas GC7 treatment decreased them. Furthermore, GC7 significantly inhibited EBV DNA copy numbers. These results confirm that active polyamine biosynthesis and high eIF5A^H^ levels contribute to EBV replication, indicating that targeting the polyamine biosynthetic pathway represents a promising therapeutic strategy against EBV infection.

Polyamines are well‐established facilitators of multiple stages of the viral lifecycle, including viral DNA replication, protein synthesis, and virion packaging. Beyond these canonical roles, polyamines also function to stabilize the left‐handed Z‐DNA conformation [36]. Cellular spermidine has been demonstrated to promote the B‐to‐Z DNA transition, which in turn attenuates cGAS activity and suppresses type I interferon responses, representing a vital immune evasion mechanism for DNA viruses including EBV [21]. Consistent with this paradigm, our work revealed that EBV upregulates host polyamine biosynthesis, which promotes the B‐to‐Z transition of viral DNA and inhibits type I IFN responses in host cells, even during lytic replication. ADAR1 and ZBP1 are the only two mammalian proteins harbouring the Zα domain that specifically binds Z‐DNA and Z‐RNA, and ZBP1 mediates protective immune responses against infection [37]. The accumulated cytosolic Z‐DNA could be specifically sensed by ZBP1, which recruits RIPK1/RIPK3 to directly phosphorylate IRF3 and activate the cGAS‐STING cascade [38]. Conversely, our recent work demonstrates that EBV‐LMP1 accelerates Ro52‐dependent K48‐linked ubiquitination and degradation of IRF3, which blocks cGAS‐STING‐IFN signaling in NPC cells [29]. Collectively, this crosstalk enables EBV to evade host innate immune surveillance and maintain persistent infection, which may also contribute to the malignant phenotype of NPC.

Interestingly, the efficacy of chemotherapeutic agents such as cisplatin also depends on their ability to activate antitumor immunity, specifically by inducing cytoplasmic DNA (cytoDNA) that triggers the STING‐IRF3‐IFN pathway [28]. Our previous work showed that EBV blocks cisplatin‐induced innate immunity by enhancing the degradation of IRF3 [29]. Here, we identify that under cisplatin treatment, EBV‐driven polyamine anabolism actively facilitates the B‐to‐Z DNA transition; this conformational change blunts subsequent innate immune signaling, ultimately leading to cisplatin resistance in EBV‐positive NPC. This resistance mechanism, based on polyamine‐mediated DNA conformational change and immune suppression, may not be exclusive to EBV‐associated malignancies. Other tumors with hyperactive polyamine metabolism could potentially employ a similar strategy to evade chemotherapeutic‐induced innate immune response and develop treatment resistance. Of note, the in vivo xenograft assays were conducted in nude mice, which lack functional T cells and intact adaptive immunity. The anti‐tumor effects we observed are attributed to innate immune activation within tumor cells, rather than a full systemic anti‐tumor immune response. Future studies with immunocompetent syngeneic models will be warranted to further verify whether ODC1 blockade improves intratumoral T cell infiltration and adaptive anti‐tumor responses.

Platinum‐based chemotherapeutics disrupt the polyamine balance in cancer cells by modulating the expression of key enzymes in the polyamine metabolic pathway: they upregulate the activity of the catabolic enzyme SSAT and, conversely, downregulate the activity of critical biosynthetic enzymes, specifically ODC1 and SAMDC. This ultimately leads to a marked depletion of the intracellular polyamine pool [23, 24]. Notably, ODC1 is highly expressed in cisplatin‐resistant ovarian cancer cells, and inhibiting polyamine uptake enhances cellular sensitivity to cisplatin [25, 26]. These findings suggest that the regulation of polyamine metabolism holds considerable therapeutic potential for improving the efficacy of platinum‐based chemotherapy. In the present study, we found that polyamine biosynthesis is significantly activated in EBV‐positive NPC cells. EBV infection is one of the key factors of chemotherapeutic efficacy. Our previous studies have demonstrated that EBV‐encoded oncogenes significantly enhance cisplatin resistance by activating multiple oncogenic signaling pathways [11, 27, 29]. Both in vitro and in vivo experiments confirmed that targeting ODC1, the rate‐limiting enzyme of polyamine biosynthesis, with DFMO effectively improves the sensitivity of EBV‐positive NPC cells to cisplatin.

In this study, we demonstrate that EBV transcriptionally activates ODC1 to augment polyamine anabolism, leading to expansion of the intracellular polyamine pool. This metabolic reprogramming concurrently fuels the spermidine‐eIF5A^H^ axis and drives Z‐DNA formation, thereby enhancing viral replication and conferring a survival advantage to NPC cells. Furthermore, we confirm that targeted inhibition of ODC1 effectively restores cisplatin sensitivity in EBV‐positive NPC.

## 4 Experimental Section/Methods

### 4.1 Cell Culture

The human NPC cell lines, HONE1, and HONE1‐EBV, were generously provided by Professor Sai Wah Tsao from the University of Hong Kong. 293T cells were cultured in DMEM medium (Cat: 11995065, Gibco, Grand Island, USA) supplemented with 10% fetal bovine serum (FBS; Cat: 04‐001‐1, BI, Kibbutz Beit‐Haemek, Israel).

### 4.2 Reagents and Antibodies

LipoMax plasmid transfection reagent (Cat. 18101223) was purchased from SUDGEN (Bellevue, WA, USA). Dynabeads (Cat. 10002D) were purchased from Thermo Fisher (Waltham, MA, USA) and IP buffer (Cat. P0013) was purchased from Beyotime (Shanghai, China). Cell Counting Kit‐8 solution (CCK8) was purchased from DOJINDO (Cat. CK04, Kyushu, Japan). Anti‐ODC1 (Cat. A3898), anti‐eIF5A (Cat. A2016), anti‐TRAF1 (Cat. A0150), anti‐Flag (Cat. AE092), anti‐mouse IgG‐HRP(Cat. AS003), and anti‐rabbit IgG‐HRP (Cat. AS014) were purchased from ABclonal. DFMO (Cat. HY‐B0744B), Doxycycline hyclate (Cat. HY‐N0565B), and GC7 (Cat. HY‐108314A). Anti‐Z‐DNA was purchased from Abcam (Cat. ab2079). Anti‐EBV Zta was purchased from Santa Cruz Biotechnology (sc‐53904). Anti‐EBV EAD was purchased from Merck Millipore (MAB8186). Anti‐Hypusine antibody was purchased from AntibodySystem (RGK08101). Anti‐ODC1 for IF and IHC (67336‐1‐Ig) and anti‐cGAS (26416‐1‐AP) were purchased from Proteintech Group.

### 4.3 Plasmids

Stable transduction of the sh‐lentiviral was performed as previously described [39]. The packaging plasmids: pMDLg/pRRE (Gag/Pol), pRSV‐Rev (Rev), and phCMV‐VSV‐G. Positive cells were selected using puromycin. shODC1 #1 sequence: GCATGTATCTGCTTGATATTG, shODC1 #2 sequence: GCTTTCACGCTTGCAGTTAAT.

### 4.4 RNA Isolation and Quantitative PCR

Total mRNA was isolated by using the NucleoZOL reagent (Cat: 740404, Macherey Nagel GmbH & Co. KG, Düren, Germany). The RevertAid First Strand cDNA Synthesis Kit (Cat: K16325, Invitrogen, Carlsbad, CA, USA) was used for reverse transcription. Quantitative PCR analysis was performed in triplicate using the SYBR Green Master Mix (Cat: A25742, Invitrogen) and the ABI7500 Real‐Time System (Applied Biosystems). All primers used in this study were listed in the Supplemental Table S1.

### 4.5 Metabolic Profiling

CNE2‐IR and CNE2 cells were collected and quickly frozen. Further sample preparation, metabolic profiling, peak identification, and curation were performed by Applied Protein Technology (APT, Shanghai).

### 4.6 Non‐Targeted Metabolic Flux

Cells(1 × 10^7^) were incubated with 20 µm ^13^C_6_‐Arginine (MCE) for 24 h at 37°C. Then cells were quickly washed with 1×PBS and fixed with a pre‐cooling buffer (methanol (HPLC‐MS grade, Millipore): H_2_O = 4:1). Then the cells were stored at ‐80°C. Further sample preparation, metabolic profiling, peak identification, and curation were performed by LipidALL Technologies as described previously [40].

### 4.7 Western Blot

Western blot analysis was conducted as previously described [39]. Visualization was performed using the ChemiDoc XRS system and Image Lab software (Bio‐Rad, CA, USA). Western blots were derived from the same experiment and processed in parallel.

### 4.8 ElisaElisa

Spermidine and IFN‐β quantification was examined using the ELISA kits (Spermidine: CEX053Ge; IFN‐β: SEA222Hu; Cloud‐Clone Corp) according to the manufacturer's instructions. Cells were washed with ice‐cold PBS and lysed in IP lysis buffer, 50 µL of cell lysate was added to each detection well, and the results were read at 450 nm.

### 4.9 Cell Proliferation Assay

Cell viability was examined using the CCK‐8 solution according to the manufacturer's instructions. The plate was read at 450 nm wavelength on a microplate reader (Biotek EL × 800, USA).

### 4.10 Chromatin Immunoprecipitation (ChIP)

ChIP assays were performed using Chromatin Immunoprecipitation Kits (Cat. 17–10085, Millipore) as previously described [21]. Anti‐cGAS (2 µg) or anti‐Z‐DNA (2 µg) was applied, and qPCR was performed using the EBV nucleic acid quantitative detection kit (DA0153, DAAN GENE, China) to assess EBV genomic DNA binding to the specified antibody. Anti‐BZLF1 (2 µg) was applied, and qPCR was performed to detect BZLF1 binding to the ODC1 promoter.

### 4.11 Clinical Specimens

The study was approved by the Medical Ethics Committee of Xiangya Hospital, Central South University (No. 201803134). Nasopharyngitis and NPC tissues were collected from the Department of Pathology at Xiangya Hospital, Central South University, Changsha, China. All clinical data were obtained from the hospital's pathologic records. The NPC tissue array (n = 140) was purchased from Outdo Biotech (NPC1401, Shanghai, China). RNA‐seq data from NPC patients were obtained from the Gene Expression Omnibus (GEO) database GSE102349. EBV activity score was calculated as previously described [41].

### 4.12 Immunohistochemistry

Immunohistochemical analysis was conducted as described previously [39]. ODC1 protein expression were determined based on staining intensity and the percentage of immunoreactive cells. The staining intensity was rated as 0 (no staining), 1 (weak staining), 2 (moderate staining), and 3 (strong staining). The percentage of immunoreactive cells was graded as 0 (no staining), 1 (<25%), 2 (25%–50%), 3 (50%–75%), and 4 (>75%). Tissue IHC scores were calculated by multiplying the intensity and the percentage of positive tumor cells. All IHC slides were evaluated blindly by two independent pathologists with no information on the clinical data provided. Slides were scanned by a Pannoramic MIDI digital slide scanner (3DHISTECH). The patients were divided into low and high expression groups according to the median scores. IHC images were acquired by a BioTek Lionheart FX automated microscope (Agilent).

### 4.13 Multiplex Immunofluorescence

Multiplex immunofluorescence of NPC tissue array was detected using Multiplex immunofluorescence staining assay kit (AFIHC025, AiFang biological, China) following the manufacturer's recommendations. The tyramide signal amplification was TSA (520‐TSA, 620‐TSA). Multispectral images were analyzed, and positive cells were quantified at a single‐cell level by inForm 2.6.0 and Phenochart 1.0.9 image analysis software.

### 4.14 Colony Formation

NPC cells were seeded at 1 × 10^3^ cells/well in six‐well plates in triplicate. After 10–14 days following indicated treatments, cells were washed with 1×PBS, fixed in methanol for 20 min, and stained with crystal violet for 15 min at room temperature. Colonies containing more than 50 cells were counted using the ImageJ software, and the survival fractions were calculated.

### 4.15 Proteomics

After cell collection, protein sample preparation, protein qualitative analysis, and results analysis were performed by Biotree Technology.

### 4.16 Tumor Xenograft Studies

All animal studies complied with ethical guidelines approved by the Institutional Animal Care and Use Committee (IACUC) of Central South University (No. 2020sydw0277). Female BALB/c‐nude mice (5–6 weeks old) were purchased from SLAC Laboratory Animal Co. Ltd. (Changsha, China). All mice were subcutaneously inoculated with HONE1‐EBV cells (5 × 10^6^ cells/mouse) in the right armpit. When the tumor volume reached 100 mm^3^ (day 9 after injection), the mice were divided randomly into 4 groups (*n* = 6 each; saline‐treated (control), DFMO‐treated, cisplatin‐treated, cisplatin and DFMO‐treated). Single‐drug treatment with DFMO (169 mg/kg) or cisplatin (4 mg/kg) was initiated by i.p. injection. The vehicle control was administered in 0.9% saline. Tumor volume and B.W. were recorded every 2 days. The tumor size was calculated as follows: tumor size = ab^2^/ 2, where a and b are the larger and smaller diameters, respectively. After 10 days of treatment, the mice were euthanized, and tumors were removed and weighed. For toxicity assessment, liver, spleen, and kidney tissues from all groups were collected, fixed, sectioned, and stained with H&E for histopathological evaluation of organ injury.

### 4.17 Luciferase Reporter Assay

Luciferase activities were detected using Dual‐Luciferase Reporter Kit (E1910, Promega) following the manufacturer's recommendations. Cells were transfected as firefly luciferase reporter gene plasmid: sea kidney luciferase reporter gene plasmid = 50:1. The results were detected by multi‐functional enzyme labeling instrument (PerkinElmer VICTORTM X3, USA). Result analysis: Ratio = (first fluorescence value background fluorescence value)/ (second fluorescence value background fluorescence value)

### 4.18 DNA Extraction and EBV DNA Copy Detection

Total DNA from cells and culture supernatant fractions was extracted using the DNA Isolation Mini Kit‐BOX 1 (DC102‐01, Vazyme), following the protocol recommended by the manufacturer. EBV DNA copies were detected using the EBV nucleic acid quantitative detection kit (DA0153, DAAN Gene), which targets the BamHI‐W region of the EBV DNA genome.

### 4.19 Immunofluorescence Staining for Z‐DNA

Immunofluorescence staining was conducted as previously described [21]. Briefly, cells were incubated overnight at 4°C with Z‐DNA antibody (Cat. ab2079, Abcam). Cells were then stained with Alexa Fluor 555‐conjugated secondary antibodies (Invitrogen). The nuclei were stained with DAPI. Images were acquired and analyzed by a BioTek Lionheart FX automated microscope (Agilent).

### 4.20 Quantification and Statistical Analysis

All statistical calculations were performed with the GraphPad Prism 9 software. The experimental data are presented as the mean value ± SD. The statistical significance of the data was analyzed using ANOVA or a standard Student's t test. Overall and disease‐free survival were determined by the Kaplan–Meier method and compared using a log‐rank test. *p* value of <0.05 was considered statistically significant.

## Author Contributions

**Yueshuo Li**: funding acquisition, investigation, conceptualization, Writing – original draft, Writing – review and editing. **Xudong Hu**: investigation, writing – original draft, writing – review and editing. **Na Liu**: investigation, validation, writing – review and editing. **Chenxing Yang**: investigation, validation, writing – review and editing. **Lin Wang**: investigation, validation, writing – review and editing. **Xi Cai**: investigation, writing – review and editing. **Min Tang**: resources, validation, writing – review and editing. **Ya Cao**: resources, writing – review and editing. **Li Shang**: conceptualization, writing – original draft, writing – review and editing, supervision, resources. **Feng Shi**: conceptualization, writing – original draft, writing – review and editing, supervision, resources, funding acquisition.

## Funding

This study was supported by the National Natural Science Foundation of China (82573064, 82303137 and 82073030) and Hunan Provincial Natural Science Foundation of China (2025JJ60499 and 2022JJ20083).

## Ethics Statement

The study was approved by the Medical Ethics Committee of Xiangya Hospital, Central South University (No. 201803134). All animal studies were performed in compliance with ethical guidelines approved by the Institutional Animal Care and Use Committee (IACUC) of Central South University (No. 2020sydw0277).

## Conflicts of Interest

The authors declare no conflicts of interest.

## Supporting information




**Supporting File**: advs77204‐sup‐0001‐SuppMat.docx.

## Data Availability

The data that support the findings of this study are available from the corresponding author upon reasonable request.
