# Supplementary material for: EBV‐Driven ODC1 Upregulation Enhances Polyamine Anabolism to Promote Viral Replication and Cisplatin Resistance in Nasopharyngeal Carcinoma
Source: Adv Sci (Weinh). 2026 Aug 15;13(61):e77204. doi: 10.1002/advs.77204 (PMC13477251; doi:10.1002/advs.77204)
Supplement: Supplementary file 1 [file ADVS-13-e77204-s001.docx]

Supplemental Table 1

| **Primers** | **Sequence** |
| --- | --- |
| ARG2-F | TCTGCTGATTGGCAAGAGACA |
| ARG2-R | GGTCCCAGGTCTAACCCCAA |
| ODC1-F | CCTGGGCGCTCTGAGAT |
| ODC1-R | GCATAAAAGGGGGTGACACG |
| SRM-F | TTGGACGGTGTCATCCAGTG |
| SRM-R | CCCCGATGATCAGCACCTTT |
| SMS-F | ACAGCACGCTCGACTTCA |
| SMS-R | TCAAATTGGCAAAGCTGCCG |
| SMOX-F | CTCAGCAGGGAAAGGTTCCTA |
| SMOX-R | TCAAGAAGTGCTTTGGCTGC |
| SAT1-F | GACGAATGAGGAACCACCTCC |
| SAT1-R | TCATATTTAGCCAGCTCCTTGAT |
| PAOX-F | CCGGAGAGTGACAGGAAACC |
| PAOX-R | GGCCACGTAGCTGTAGGAC |
| BMRF1-F | CTAGCCGTCCTGTCCAAGTGC |
| BMRF1-R | AGCCAAACGCTCCTTGCCCA |
| IFNβ-F | ACGCCGCATTGACCATCTAT |
| IFNβ-R | GTCTCATTCCAGCCAGTGCTA |

Supplemental Table 2

| **Position** | **Plasmids** | **Sequence** |
| --- | --- | --- |
| 331-350 | EAD-mut | TVSPSPS**RLLRL**RTPTWESP |
| 331-350 | EAD-wt | TVSPSPS**PPPPP**RTPTWESP |

**Supplemental Figure 1**

A, Pie charts indicating the number of biochemical changes of each metabolic pathway in HONE1-EBV cells compared with HONE1 cells. B, NPC cells were incubated in ^13^C_6_-arginine (20 μM) for 24 hours. Metabolites were extracted and analyzed as described in Materials and Methods. C-D, Relative metabolite abundance of metabolites from polyamine-targeted metabolomics analysis. E-F, mRNA expression of the main polyamine metabolic enzymes in HONE1/ HONE1-EBV (left) and CNE2/ CNE2-EBV cells (right) was detected by qRT-PCR (n=3).

**Supplemental Figure 2**

ODC1 expression levels were calculated relative to EBV gene expression. Groups with high and low EBV levels were defined according to the median expression from GSE102349.

**Supplemental Figure 3**

A, Spermidine levels in HONE1-EBV cells were measured by ELISA with TPA (10 μM) treatment at the indicated time points. B, HONE1 and CNE2 cells were transfected with empty vector (Vector) or BZLF1-expressing plasmids in different doses, and cell lysates were subjected to Western blot analysis.

**Supplemental Figure 4**

A-B, Real-time PCR showing EBV DNA copy numbers in HONE1-EBV and CNE2-EBV cells infected with ODC1 shRNA plasmids. C, Real-time PCR showing EBV DNA copy numbers in ODC1-knockdown NPC cells following Doxy (10uM, 24 hours) treatment. D, NPC cells were pretreated with DFMO for 24 hours, followed by treatment with Doxy (10uM) for an additional 24 hours. EBV DNA copy numbers were quantified by real-time PCR. E, Representative IHC-F staining of ODC1 and EAD expression in a tissue microarray of adjacent noncancerous tissues (ANT) and NPC (Scale bar: 50 μM). F, ODC1 expression levels were quantified relative to EAD expression. High and low ODC1 expression groups were defined according to the median expression value. G, The expression levels of *IFNB* mRNA with the indicated treatments were measured by real-time PCR, NC: negative control.

**Supplemental Figure 5**

A, HONE1-EBV and CNE2-EBV cells were transfected with plasmids, and cell viability was assessed using the CCK-8 assay at the indicated time points. B, HONE1-EBV and CNE2-EBV cells were treated with or without DFMO (10 μM), and cell viability was assessed using the CCK-8 assay at the indicated time points. C-D, EdU staining analysis of CNE2-EBV cells treated with shODC1 or DFMO. EdU fluorescence intensity with an average value above 500 was defined as positive. Nuclei were counterstained with Hoechst. (Scale bar: 200 μM). E, ODC1 knockdown CNE2-EBV cells were treated with or without spermidine (1 mM), and cell viability was assessed using the CCK-8 assay at the indicated time points. F, CNE2-EBV cells were treated with or without DFMO (10 μM) for 24 hours, followed by incubation with spermidine (1 mM), and cell viability was assessed using the CCK-8 assay at the indicated time points. G, CNE2-EBV cells were treated with spermidine (1 mM) alone or spermidine combined with GC7 (10 μM), and cell viability was assessed by the CCK-8 assay at the indicated time points. H, Cell viability was assessed by the CCK-8 assay following the indicated treatments. Error bars, SD.

**Supplemental Figure 6**

IHC staining of IgG as negative control in pathological sections from NPC patients (Scale bar: 100 μM)

**Supplemental Figure 7**

A, ODC1 knockdown CNE2-EBV cells were treated with or without cisplatin (10 μM), and cell proliferation was assessed using the colony formation assay. Relative survival fractions are shown. B, CNE2-EBV cells were treated with or without DFMO (10 μM) for 24 hours, followed by incubation with or without cisplatin (10 μM). Cell proliferation was assessed using the colony formation assay. Relative survival fractions are shown. C and E, The combination index (CI) of DFMO and cisplatin was calculated by experimental Chou-Talalay equations. fa represents the cell inhibition rate at the corresponding drug concentration; CI<1 indicates a synergistic effect, CI>1 indicates an antagonistic effect, and CI=1 indicates an additive effect. D, CNE2-EBV cells were treated with or without DFMO (10 μM) for 24 hours, followed by incubation with or without cisplatin (10 μM). Cell viability was assessed using the CCK-8 assay.

**Supplemental Figure 8**

A, MDA-MB-231 cells were stained for Z-DNA after the indicated treatments. cisplatin: 10 μM, DFMO: 10 μM, spermidine (Spd): 10 μM. Scale bars: 20 μM. B-C, Quantification of Z-DNA fluorescence signal intensity. D-E, The production of IFN-β protein was assayed by ELISA. The relative production of IFN-β was calculated.

**Supplemental Figure 9**

The NPC xenograft model was established using HONE1-EBV cells. A-B, The tumor volumes of xenograft tumors (A),and the body weight of mice (B) following various treatments. Error bars, SD. C, H&E staining on liver, spleen, and kidney tissues from all groups.Scale bar: 100 μM
